# Metasurface with all-optical tunability for spatially-resolved and multilevel thermal radiation

**DOI:** 10.1515/nanoph-2024-0005

**Published:** 2024-03-13

**Authors:** Shuhui Jiao, Kang Zhao, Jianhui Jiang, Kailin Zhao, Qin Guo, Jingbo Wang, Yansong Zhang, Gang Chen, Qian Cheng, Pei Zuo, Weina Han

**Affiliations:** Laser Micro/Nano-Fabrication Laboratory, School of Mechanical Engineering, 47833 Beijing Institute of Technology, Beijing 100081, China; Beijing Institute of Technology Chongqing Innovation Center, Chongqing 401120, China; School of Mechanical and Electrical Engineering, 34756 Wuhan Institute of Technology, Wuhan 430073, China

**Keywords:** phase-change materials, ultrafast laser, dynamic modulation, multilevel infrared camouflage, visible-infrared compatible information storage

## Abstract

Manipulating the thermal emission in the infrared (IR) range significantly impacts both fundamental scientific research and various technological applications, including IR thermal camouflage, information encryption, and radiative cooling. While prior research has put forth numerous materials and structures for these objectives, the significant challenge lies in attaining spatially resolved and dynamically multilevel control over their thermal emissions. In this study, a one-step ultrafast laser writing technique is experimentally demonstrated to achieve position-selective control over thermal emission based on the phase-change material Ge_2_Sb_2_Te_5_ (GST). Ultrafast laser writing technique enables direct fabrication and manipulation of laser-induced crystalline micro/nano-structures on GST films. Thermal emission can be precisely controlled by adjusting the pulse energy of the ultrafast laser, achieving a high thermal emissivity modulation precision of 0.0014. By controlling thermal emission, the ultrafast laser writing technique enables multilevel patterned processing. This provides a promising approach for multilevel IR thermal camouflage, which is demonstrated with emissivity-modulated GST emitters. Remarkably, ultrafast laser-induced crystalline micro/nano-structures display geometric grating features, resulting in a diffraction-based structural color effect. This study demonstrates the effective use of laser-printed patterns for storing information in both visible and infrared spectrum.

## 1 Introduction

The manipulation of the thermal emission is essential for thermal photonic devices, with applications spanning thermal camouflage [1], [2], thermal management [3], [4], and thermal infrared sources [5], [6]. Thermal emission refers to the outflow of radiative electromagnetic energy caused by the thermal motion of charged particles [7]. Determined by Planck’s law of blackbody radiation, the thermal emission of a material depends on its thermal emissivity (*ε*) and actual temperature (*T*). In thermal camouflage, an object’s radiative temperature is adjusted to match the background temperature by controlling thermal emission, especially emissivity [8]. Consequently, captured by a thermal infrared imager which distinguishes the differences in thermal radiation, the object can be hidden in the background. Traditional thermal camouflage involves applying a low-emissivity coating to the object’s surface to reduce its radiative temperature, thereby achieving thermal camouflage [9], [10]. Nonetheless, traditional coatings have a fixed emissivity, rendering this camouflage method static, and objects covered by it can only blend in with backgrounds at particular temperatures. If the background temperature changes, variations in radiative temperatures between the object and the background can lead to easy detection by a thermal infrared imager. Hence, dynamically adjusting the object’s emissivity is crucial for promoting the development and application of IR field.

The dynamic modulation of thermal emissivity has been widely explored using stimuli-responsive active materials with variable optical and thermal characteristics, including semiconductors [11], [12], graphene [1], [2], thermochromic metamaterials [13], [14], phase-transition materials (PTM) [1], [15], and phase-change materials (PCM) [10], [16], [17], among others. Among candidate materials, chalcogenide phase-change materials, especially Ge_2_Sb_2_Te_5_ (GST) [18], emerge as promising choices for enabling dynamic modulation functionality. Notably, GST displays markedly different characteristics in its amorphous and crystalline phases, with phase switching achievable through thermal sources [19], electrical pulses [20], and laser pulses [21]. Earlier research has demonstrated that an IR thermal emitter, consisting of a GST film overlying a metal film, can continuously adjust thermal emissivity by modulating the phase state proportions of the GST thin film through heat treatment [16], [22]. Nonetheless, even though switchable spectral control of thermal emissivity has been accomplished, the difficulties persist in achieving multi-level [23], [24], position-selective and continuous control of thermal radiation, primarily due to processing technique limitations. Lately, employing a photomask presents a solution to this problem [25], [26]. For example, a study has been reported on a laser printing method to spatially tune the thermal emission [26]. They used a pulsed laser beam modulated by specific photomasks to generate position-selective patterns. And the multilevel thermal emissivity was achieved by a multiple crystallization of GST in a layer-by-layer manner. Although spatial and spectral processing is feasible, the use of a photomask leads to high costs and limited precision. Therefore, using this method to achieve spatial and continuous control of thermal emission is difficult. As a result, achieving spatially resolved, dynamically reconfigurable control of thermal emission for practical applications remains a challenging endeavor.

Herein, a one-step ultrafast laser writing technique is experimentally proposed to position-selectively and multilevel-dynamically control the thermal emission of a GST thermal emitter. Crystallization-induced multilevel emission patterns are directly recorded into an amorphous GST film on a metal reflector, where the crystallinity of GST is accurately controlled by the ultrafast laser. Dynamical modulation of the thermal emissivity can be achieved by the secondary irradiation of the ultrafast laser. Meanwhile, the changes in the GST properties lead to the formation of surface structure during the interaction between ultrafast laser and phase-change material, which results in a structural color display in the visible spectrum. We demonstrate a multilevel infrared camouflage based on spatially modulated GST emitters under different environment temperatures. Furthermore, the volume shrink effect is accompanied by the crystallization phase-change. Thus, a dynamically tunable metasurface compatible with both visible and infrared spectra is achieved by controlling the phase states and geometric structures of the modified pattern. Our concept provides an integrated approach for multi-functionality, especially for cross-band programmable dynamic metasurface devices.

## 2 Results and discussion

### 2.1 Design of a reconfigurable metasurface for both visible and infrared spectra with spatial and spectral modulation capability

**Figure 1:**
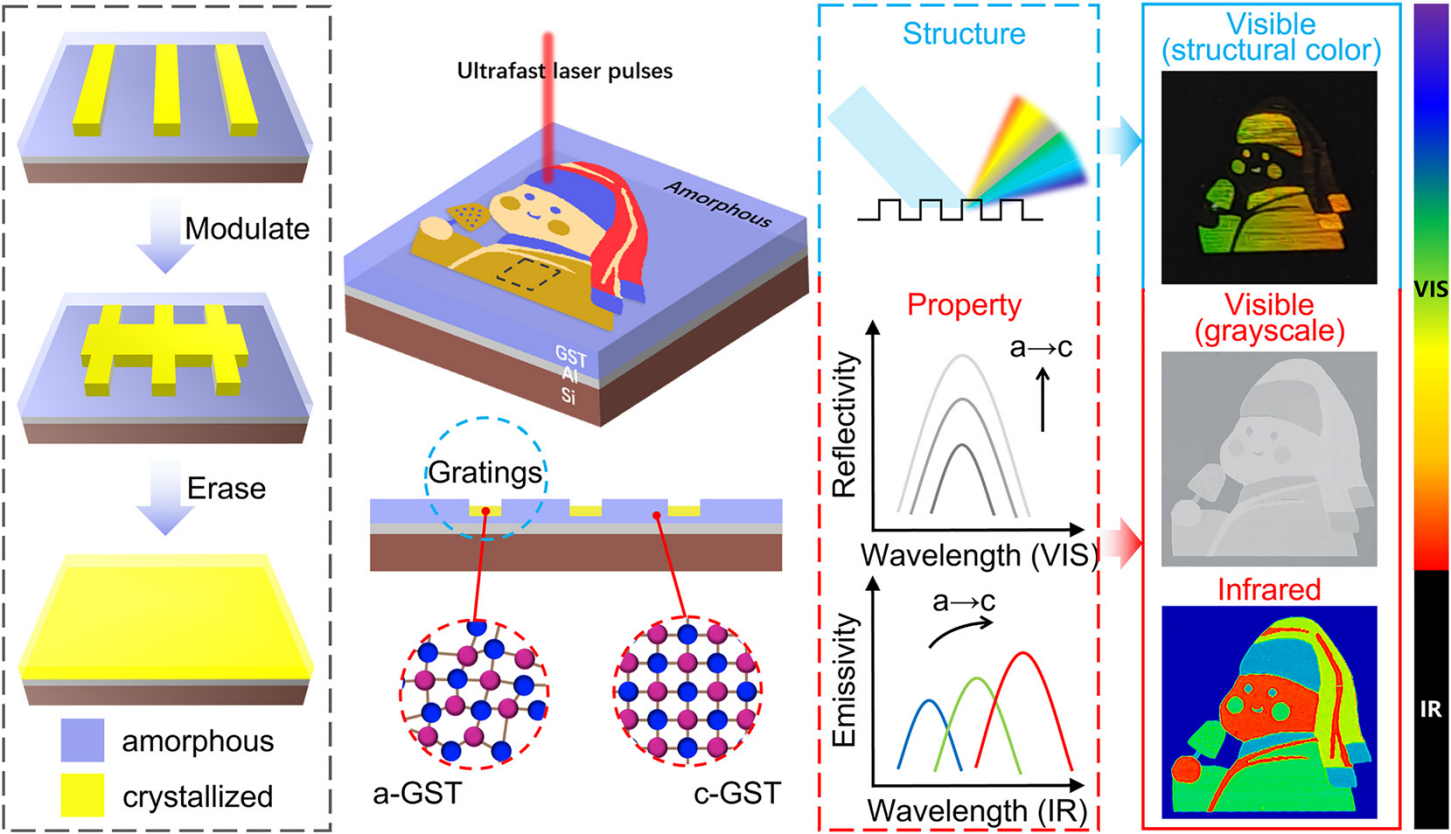
Optically reconfigurable metasurface with spatial and spectral modulation in the visible and IR wavelengths.

Figure 1 illustrates a visible-infrared-compatible dynamic metasurface scheme that utilizes ultrafast laser-induced modulation of GST properties and structure. Figure 1 demonstrates a dynamic modulation approach comprising three key steps. Firstly, ultrafast laser directly writes GST-modified nanogratings on the sample surface without the need for a photomask, forming the fundamental unit. Secondly, the nanograting structure ratio is modulated by a secondary ultrafast laser irradiation process, building upon the initial modified nanogratings to enable dynamic refreshing. Ultimately, the altered structure was completely erased through thermal annealing, resulting in full crystallization. The metasurface comprises two layers on a silicon substrate: a 350-nm-thick amorphous GST (a-GST) active layer and a 100-nm-thick Al reflector layer, arranged from top to bottom. Figure S1 displays the selection process of GST layer. GST represents a typical phase-change material for infrared applications [27]. In the mid-infrared band, crystalline GST (c-GST) exhibits a high real part of the dielectric constant (refractive index) and a relatively low imaginary part (loss), while amorphous GST experiences negligible loss [28]. The GST thickness is selected to match the response band (8−14 μm) of the long-wavelength infrared (LWIR) camera used in our experiment, which falls within the atmospheric window for infrared radiation [8]. The thickness of the Al layer effectively prevents the penetration of infrared light, rendering the substrate material optically inactive. When the ultrafast laser is applied to the surface of the sample, the phase-change occurs in the processing area with property modification driven by the crystallization threshold effect [28]. Simultaneously, the crystallization process results in the formation of a surface geometric grating structure due to volume shrinkage effects [29], [30]. Ultrafast lasers enable flexible, high-resolution pattern processing [31], [32], [33], allowing simultaneous encoding of visible structural color, gray information, and infrared signature within the same pattern through modulation of both structures and properties. Structural color arises from the diffraction effects of 2D gratings. Grayscale and infrared imaging depend on property differences in visible reflectivity and infrared emissivity before and after the GST phase-change. Encoding complex information in both visible and IR bands enables additional applications, including information security, encryption, and anti-counterfeiting.

### 2.2 Optical fabrication and modulation of the visible-infrared-compatible metasurface based on ultrafast laser-induced modified ripple structures

**Figure 2:**
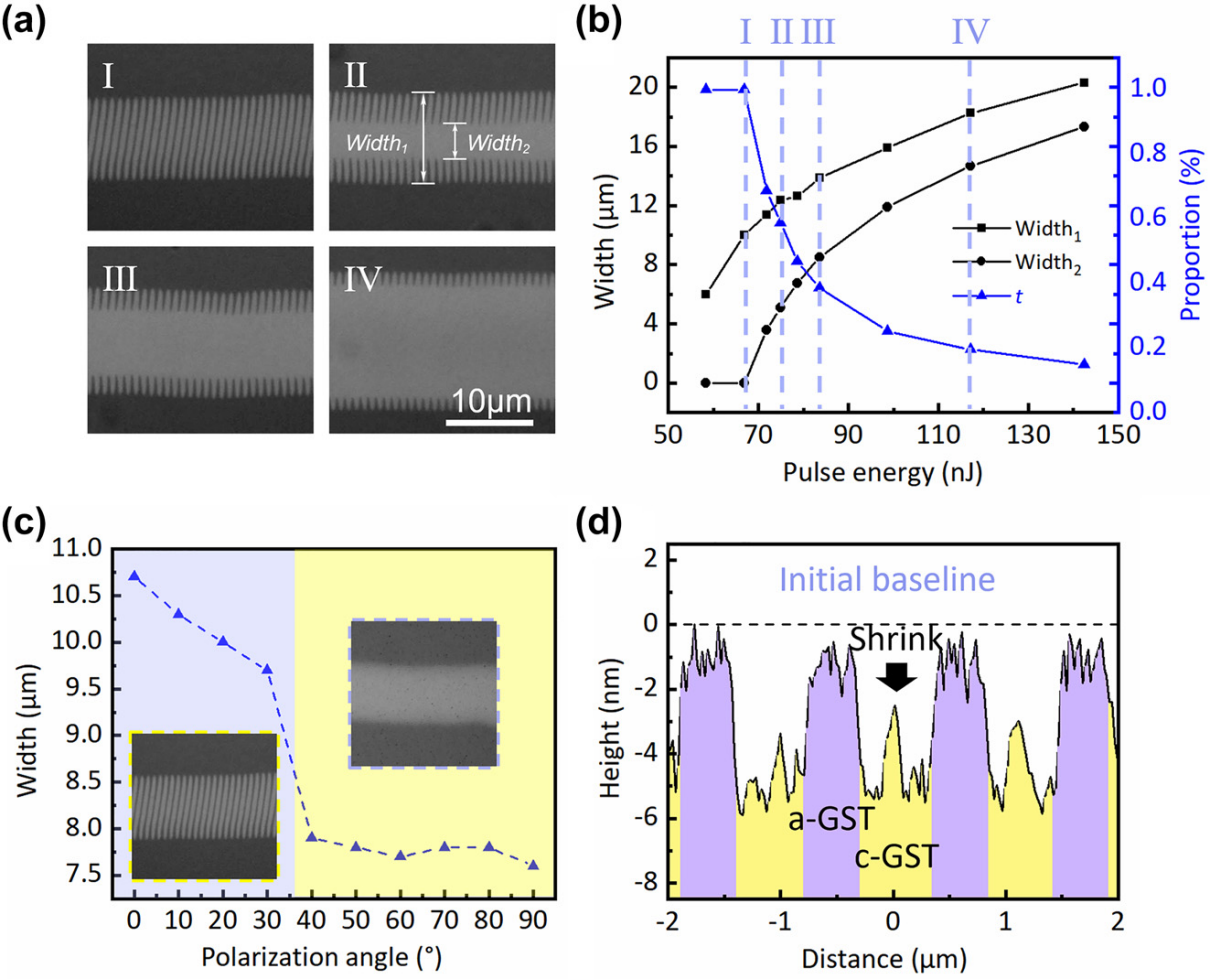
Structural characteristics of ultrafast laser-induced modified ripple structures. (a) Optical microscopy images of four ps-laser-induced GST surface structures with increased pulse energies: I (68.4 nJ), II (74.9 nJ), III (83.6 nJ), and IV (117.2 nJ). (b) The width of the scanned modified line, the width of the central fully crystallized line, and the proportion (*t*) of the modified ripple structure as a function of the irradiated pulse energies. (c) The width of the scanned modified line evolves with laser polarization angles. The illustrations are the corresponding morphologic images. (d) The AFM scanning results of the center of the modified ripple structures along the scan direction.

During laser processing, the interaction between the laser and surface plasmon polaritons (SPPs) can control the electromagnetic field distribution, enabling the fabrication of micro/nano structures on the surface [34]. Laser-induced periodic surface structures (LIPSS) primarily result from the interplay between laser processing parameters and the feedback effects of existing surface structures [35]. In this study, we create controlled surface micro/nano structures on a GST film by adjusting pulse energy to modulate laser-SPP interference. Figure 2a shows four ps-laser-induced surface structures with varying grating proportions. The pulse energies used were 68.4, 74.9, 83.6, and 117.2 nJ, respectively. At high pulse energies, the central region of the Gaussian laser beam deposits more energy into the GST film. In fact, one can observe that the widths of each area change with pulse energies, and the proportion of modified ripple structures decreases as pulse energies increase. To numerically represent this ratio, we define a parameter ‘*t*’ (modified ripple structure proportion) as follows: *t* = [(Width_1_ − Width_2_)/Width_1_] × 100 %, where Width_1_ is the line width of the scanned line, and Width_2_ is the line width of the central fully crystallized structure. Figure 2b illustrates the dimensions and proportion of modified ripple structures in relation to irradiated pulse energies. It is evident that the dimensions (Width_1_ and Width_2_) increase with rising pulse energies. The parameter ‘*t*’ decreases from 100 % to 14.8 % as the pulse energy increases, indicating a reduction in the proportion of modified ripple structures within the intermediate modified ripple structures.

The morphology of the modified structures could be controlled by adjusting the polarization parameters of the laser. To simplify the description, we define the angle between the laser polarization and the writing direction as the polarization angle. As depicted in Figure 2c, at small laser polarization angles (0–30°), the grating’s line width slightly decreases with increasing polarization angles. However, when the polarization angle exceeds 30°, the modified structure is a pure crystalline line without a grating structure, and the width of the pure crystalline line remains stable. This phenomenon is speculated to relate to the impact of SPP propagation direction and presequence structure. When the laser polarization was parallel to the processing direction, an SPP propagation direction structure existed, resulting in the subsequent formation of modified nanogratings. Conversely, when the laser polarization was at a particular angle to the processing direction, there was no predefined structure in the propagation direction of the SPP. Hence, subsequent modified nanogratings could not be formed. Two typical grating structures are depicted in illustrations (Figure 2c) with laser polarizations of 0° and 90°, respectively.

We further investigated the crystal-phase distribution of the modified nanogratings. Figure 2d shows the AFM test results along the print direction at the center of the modified nanogratings. To facilitate visual interpretation, various crystal phases were color-coded. The periodic distribution of laser energy induced a periodic crystal-amorphous fringe distribution on the sample surface. The period of this pattern corresponds to the laser wavelength and is consistent with the LSFL-I mechanism formed by LIPSS. Although the formation of modified nanogratings does not involve any material removal, the lattice arrangement discrepancy results in a decrease in the GST volume during the crystallization process. Consequently, the periodic phase distribution results in a periodic structure. A crystallization depth of 100 nm shrinks the GST surface by approximately 7 nm, and the period of alternating high and low is approximately 1.1 μm. The height information of the modified grating is visible in the AFM test results. This is the root cause of the structural color rendering.

**Figure 3:**
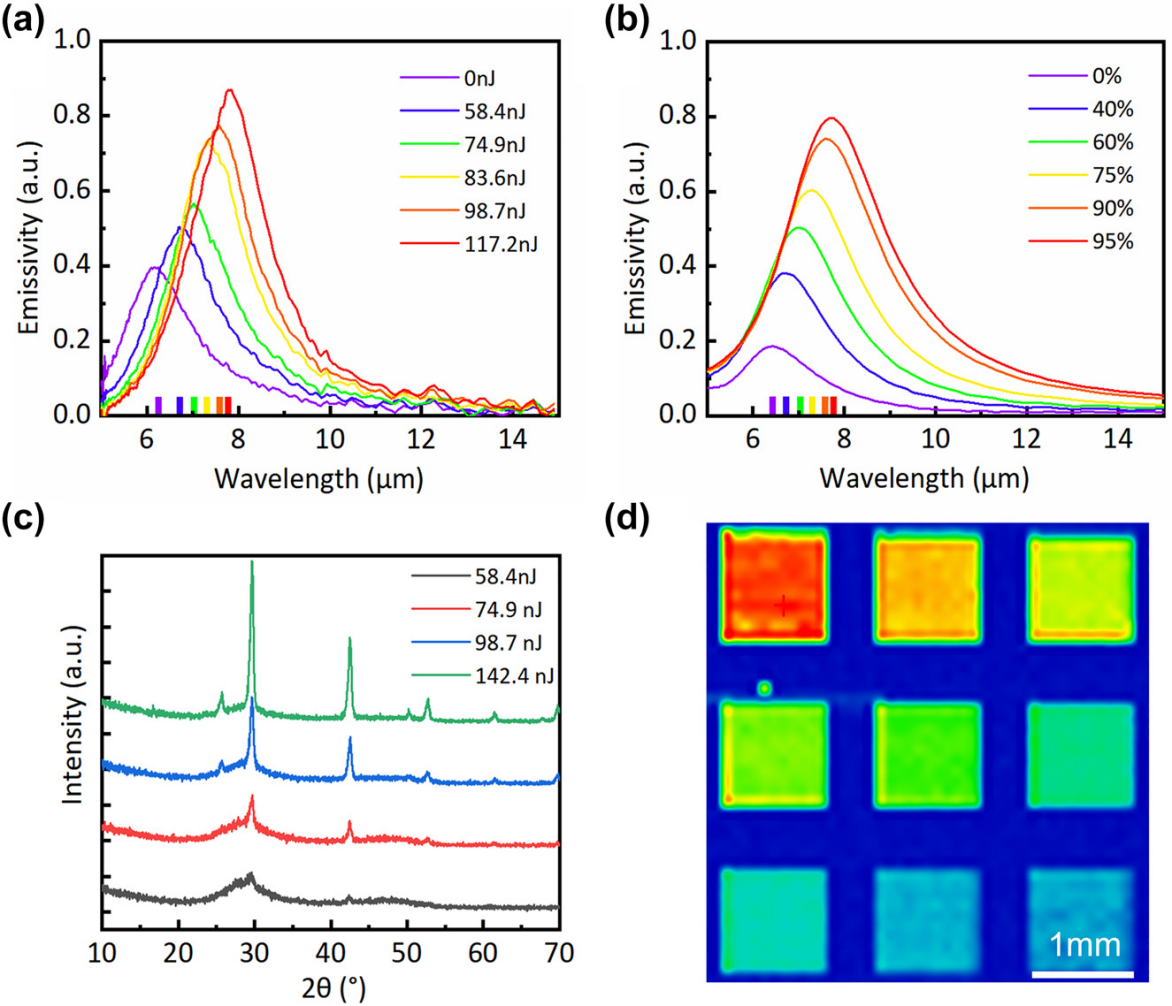
Property characteristics of ultrafast laser-induced modified ripple structures. (a) Measured emissivity spectra of samples with pulse energies of 0, 58.4, 74.9, 83.6, 98.7, and 117.2 nJ, respectively. (b) Emissivity spectra calculated using FDTD simulation. (c) Gazing incidence X-ray diffraction patterns of samples with the pulse energy as 58.4, 74.9, 98.7 and 142.4 nJ, respectively. (d) Thermal images of the experimental samples.

The pulse energy of the laser directly determines the equivalent crystallinity of the micro/nano units, which has an important effect on its emissivity spectrum in the infrared band. The influence of various energy parameters on the emissivity of the device was explored. According to the peak position and peak intensity, five optimal energy parameters were selected as the laser energy processing parameters of the final infrared multilevel camouflage device. The pulse energies were 58.4, 74.9, 83.6, 98.7, and 117.2 nJ, respectively. Figure 3a shows the emissivity spectral lines of the unprocessed region and the five groups of processed regions. As can be seen from the figure, when the laser energy is 0 nJ, that is, the device surface is now amorphous GST, and its peak value is about 0.5. With the increase of laser energy, the peak value gradually increases to 0.97. In an ideal situation, the emissivity can be continuously adjusted by precisely adjusting the pulse energy. In addition to the peak value, the peak position also redshifts with the increase of laser energy.

The influence of the crystallinity of GST films on the spectral modulation of the entire multilayer film structure is studied, as shown in Figure 3b. The Lorentz–Lorenz formula was used to obtain the dielectric constant of GST under different crystallinity (Figure S2), which was input into the material library of the simulation software for simulation. And the spectral influence brought by crystallinity was explored. It can be seen that, with the increase of crystallinity, the peak position of the emission rate is redshifted, and the peak intensity of the emission rate increases. Due to the change in the crystallinity of GST, the spatial distribution positions of its atoms are changed, which leads to the different permittivity of GST under different crystallinity. The imaginary part of the permittivity of a-GST is negligible (almost 0), while the imaginary part of the permittivity of c-GST increases with the increase of wavelength, resulting in its strong absorption capacity in the mid-infrared band. With the increase of crystallinity, the imaginary part of the dielectric constant also increases. So the ability to absorb electromagnetic waves becomes stronger, and the emission rate of the whole multilayer structure increases.

To study and verify the change law of material properties of phase-change micro/nano units under different laser energies, X-ray diffraction patterns (XRD) of phase-change micro/nano units induced by different pulse energies were measured in this study, as shown in Figure 3c. It can be observed from the figure that the characteristic peaks of (111), (200), (220), (311), and (222) appear in the XRD diffraction pattern of the phase-change micro/nano unit, which are all characteristic peaks of face-centered cube (FCC) structure [36]. With the increase of laser energy, the diffraction intensity of (200) and (220) is significantly enhanced, which is the most obvious characteristic peak of the face-centered cubic structure. When the laser pulse energy increases to 98.7 nJ, the characteristic peaks of (111), (311), and (222) appear in the XRD pattern of the phase-change micro/nano unit, and the peak intensity also shows a trend of continuous increase with the further increase of the energy. These results indicate that with the increase of laser energy, the crystallinity of phase-change micro/nano units gradually increases, and the proportion of crystalline structure in GST materials increases. It can be seen that the GST material properties can be controlled by adjusting the pulse energy.

By changing the pulse energy of the ultrafast laser, a 0.1 mm^2^ square region consisting of different types of phase-change micro/nano units is processed on the sample. Figure 3d shows its emission intensity distribution at a wavelength of 8 μm. The pulse energy decreases successively from Region 1 to Region 9, and the background is the amorphous GST surface without laser processing. Due to the increase of pulse energy, the crystallinity in the unit area increases, which leads to the increase of the emission intensity.

The radiation temperature detected by the infrared thermal imager is determined by the emissivity of the object, the actual temperature of the object and the spectral range of the infrared thermal imager (8–14 μm). Modulation of the emission peak value and position can be achieved by inducing crystallinity of different phase-change micro/nano units by ultrafast laser. Therefore, dynamic modulation of the object’s radiation temperature can be achieved by controlling the average emission in 8–14 μm band. And the application of infrared thermal camouflage with flexible matching environmental background temperatures can be realized.

### 2.3 Optical fabrication and modulation of GST modified gratings

**Figure 4:**
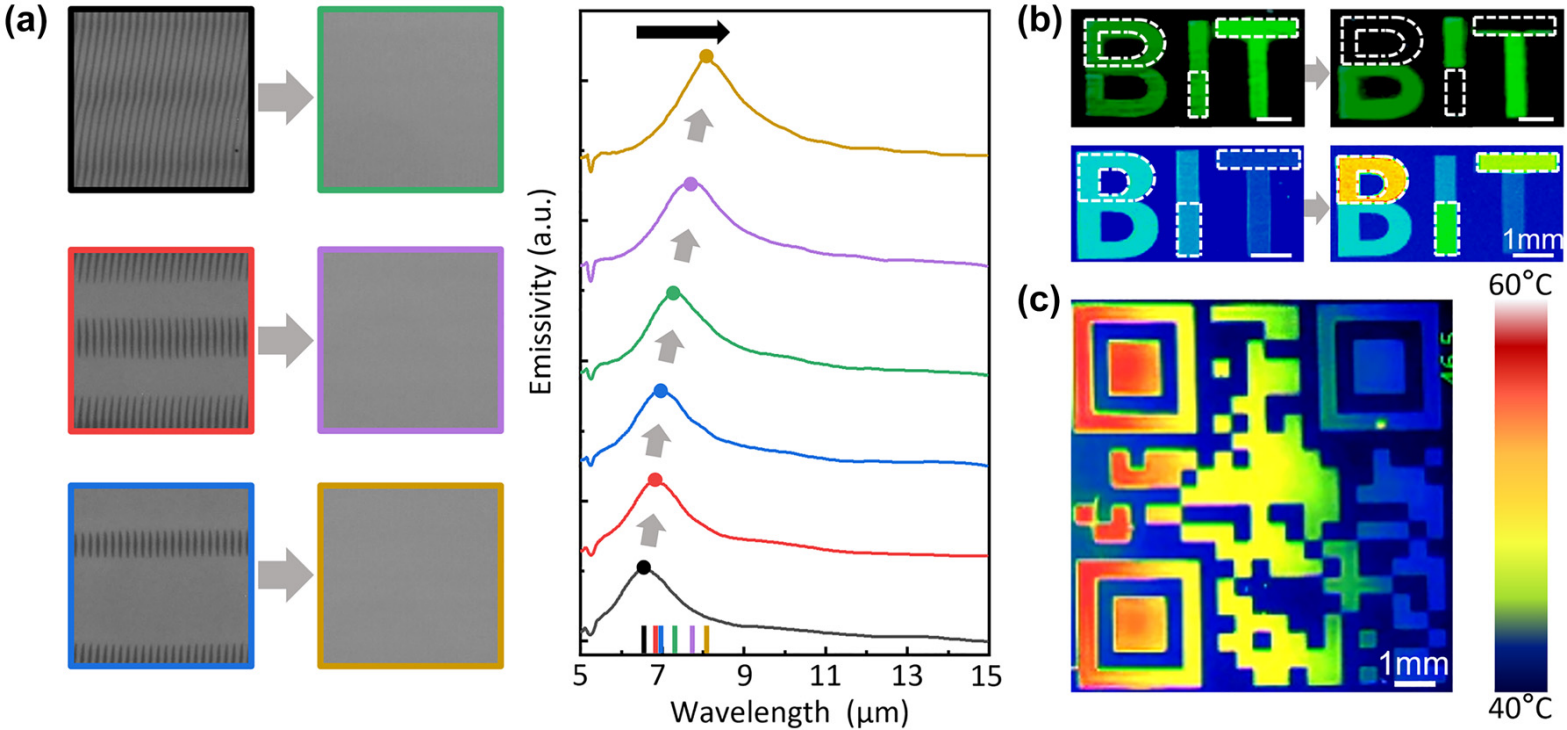
Dynamic modulation of thermal emission in space and spectrum. (a) Measured emittance spectra of modulated arrays with varying pulse energies (shifted on the *y*-axis for clarity). Light micrographs are shown on the left. (b) Top: images taken by the camera under intense light exposure before and after the modulation. Bottom: infrared spectral images before and after the modulation. (c) A continuous thermal emission pattern written onto GST emitter.

Ultrafast laser secondary modulation alters the structure and properties of the original phase-change micro/nano units, consequently influencing spectrum modulation. The laser processes the modified grating structure with a bus width equal to the structure’s sweep distance. Subsequently, a laser pulse energy of 136.8 nJ was used to perform the secondary scan on the three types of GST modified gratings. The original modified grating structure was erased and transformed into a fully crystalline structure. Simultaneously, Figure 4a’s infrared emission spectral line demonstrates that GST’s infrared emission rate redshifts as the number of scans increases. Ultrafast laser secondary modulation achieves secondary modulation of both visible and infrared band image information. Figure 4b (top) displays the visible light structural color image under intense illumination, where colors are observed due to the dispersion effect of grating diffraction. After the secondary modulation, it is evident that the upper parts of the “B”, the lower section of the “I”, and the upper area of the “T” structure do not exhibit any color. This occurs because the initial structure of the modified gratings has been replaced with a fully crystalline structure, eliminating the geometrical grating diffraction effect. Figure 4b (bottom) displays infrared imaging based on the infrared emission intensity of the “BIT” pattern at an 8-μm wavelength. Following the secondary modulation, the infrared absorptivity gradually rises with the number of scanning cycles, a consequence of heightened crystallization in GST material. In summary, ultrafast laser secondary scanning processing allows for the modulation of the structures and properties of the original phase-change micro/nano units. This process also enables the secondary modulation of visible and infrared spectra, achieving dynamic modulation of multi-band compatibility information. Furthermore, Figure 4c depicts a thermal emission pattern with continuous emission intensity, revealing the capability for continuous multi-stage processing without the need for a mask. This is achieved through precise control of laser pulse energy, and the minimum adjustment accuracy can reach 0.0014 (Figure S3).

### 2.4 Multilevel infrared camouflage under varying background temperatures

**Figure 5:**
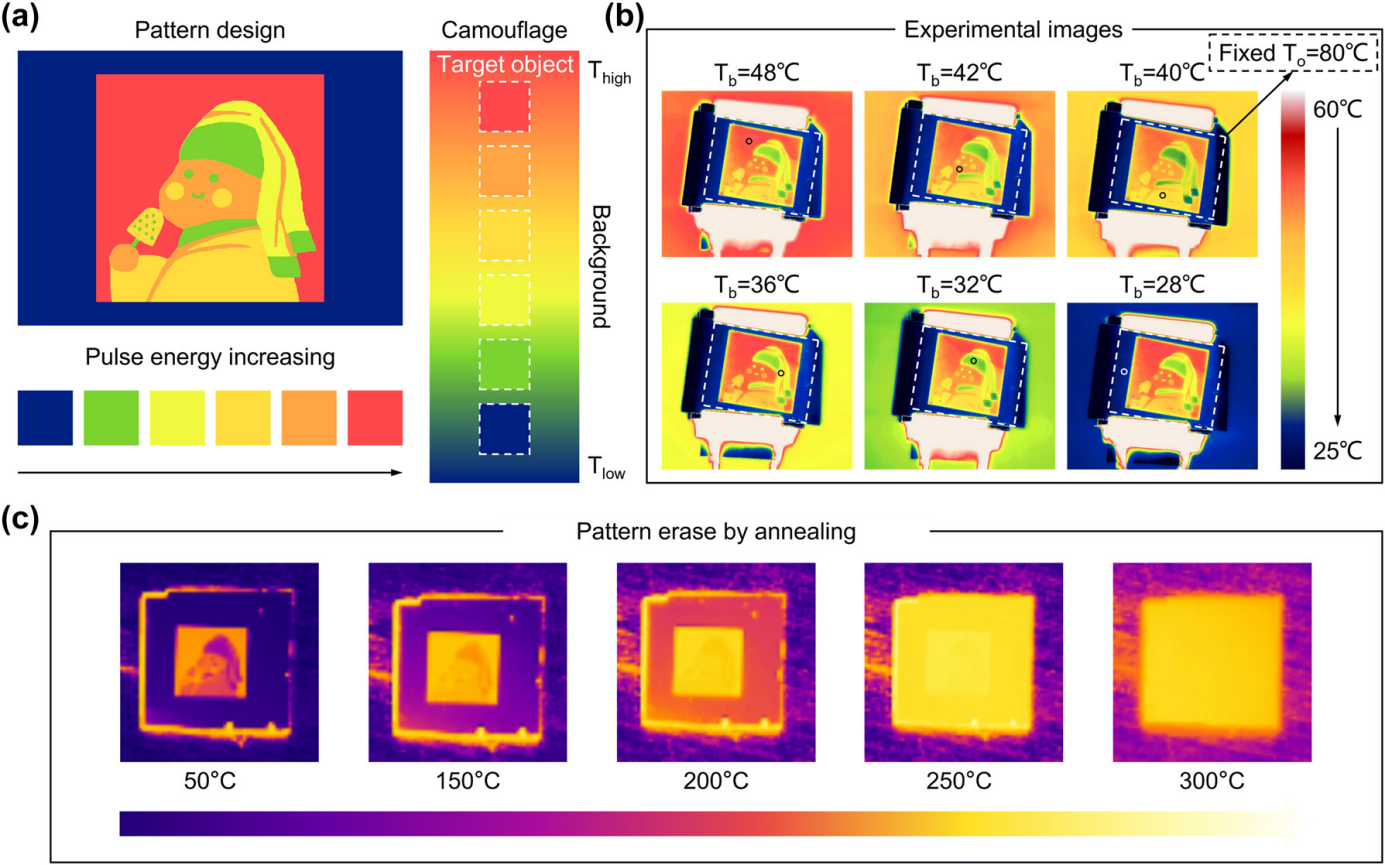
Infrared thermal selectivity for varying background temperatures. (a) Design of multilevel infrared camouflage. (b) Thermal images of a 80 °C object under different background temperature at 48, 42, 40, 36, 32, and 28 °C. (c) Annealing process of erasing the thermal pattern.

Direct laser writing of multilevel thermal patterns provides a promising platform for IR camouflage. According to the Stefan-Boltzmann law, the thermal emission (*E*) of the actual object is given by *E* = *εσT* ^*4*^, where *σ* is the Stefan–Boltzmann constant, *ε* is the emissivity of the object, and *T* is the surface temperature of the object in Kelvin. To achieve thermal camouflage for the object against the background, it’s essential to match their thermal emissions. The thermal emission is detected by an IR camera, which depends on the actual surface temperature and the average emissivity at the detecting band (8–14 μm) of the object. As far as we know, traditional thermal camouflage coatings usually have a single emissivity, while our multilevel emissivity modulation offers an effective solution to this problem. Figure 5a illustrates the mechanism for multilevel infrared camouflage against backgrounds of different temperatures. The color of the object, as captured by the IR camera, is contingent on its thermal emissions, which, in turn, rely on its surface emissivity at the same temperature. This emissivity is modulated by utilizing laser pulse energy to achieve varying crystallinity. At backgrounds of different temperatures, the multilevel thermal emissions of the object can be adjusted to match the background’s emissions, creating a consistent IR appearance that camouflages the object within the background. In our camouflage experiment, the sample is affixed to a micro heater with a constant power supply, and the background temperature is adjusted using a heating stage. Figure S4 shows the experimental device used to simulate infrared imaging of the target object under different background temperatures.

Figure 5b displays the changes in thermal images while maintaining a constant object temperature (*T*_*o*_) at 80 °C, while the background temperature (*T*_*b*_) varies from 48 °C to 28 °C. The thermal images of the device with *T*_*o*_ at 30 °C, 50 °C, 70 °C, and 90 °C are shown in Figure S5. The thermal images were captured using an IR camera while the background was cooled. *T*_*o*_ and *T*_*b*_ were measured using a thermometer. The pattern of a little girl is composed of six levels including the a-GST background. Utilizing the mentioned five levels of pulse energy, a six-level pattern is generated using ultrafast laser direct writing technology to achieve multilevel infrared thermal camouflage across varying background temperatures. The region highlighted by a circle on the processed sample exhibits localized selective camouflage in response to variations in background temperatures. At a background temperature of 48 °C, the area marked by the circle in the processed sample (generated with a pulse energy of 117.2 nJ) exhibits a higher average surface emissivity. As observed through the infrared thermal imager, this region exhibits a radiation temperature of 48 °C, matching the preset temperature. In contrast, other areas with lower average emissivity show radiation temperatures below 48 °C. With the background temperature gradually decreasing to 32 °C, the surface radiation temperature in the area indicated by the circle sequentially aligns with the background temperatures. In contrast, radiation temperatures in other regions, due to their varying average emissivity, are either higher or lower than the background temperatures. At a background temperature of 28 °C, the radiation temperature in unprocessed areas matches the background temperatures, while the radiation temperature in all processed areas higher than the background temperatures. In conclusion, this study showcases an infrared multilevel thermal camouflage device capable of operating across six different background temperatures within a variable temperature range of up to 20 °C. Moreover, it allows for dynamic infrared multilevel camouflage by multiple irradiation of ultrafast laser (shown in Figure S6). Additionally, the metasurface device holds the potential for use in flexible wearable thermal camouflage applications, including infrared stealth clothing. Figure S7 displays the IR performance of a GST flexible device.

Phase-change material GST has the potential to reversibly alter its optical properties through specific external stimuli. In our research, we initiate the removal of IR-printed images through thermal annealing. Figure 5c illustrates the step-by-step progression of erasing the thermal pattern as observed by an IR camera. The entire sample is placed on a heating stage, and we incrementally raise the temperature from 50 °C to 300 °C to induce crystallization. The pattern includes a modified surface structure, and the previously amorphous background gradually becomes fully crystallized. Notably, as the temperature reaches 300 °C, the entire sample exhibits uniform thermal emissivity, effectively erasing the multilevel thermal pattern.

### 2.5 Anti-counterfeiting with compatibility in both the visible and infrared spectra

**Figure 6:**
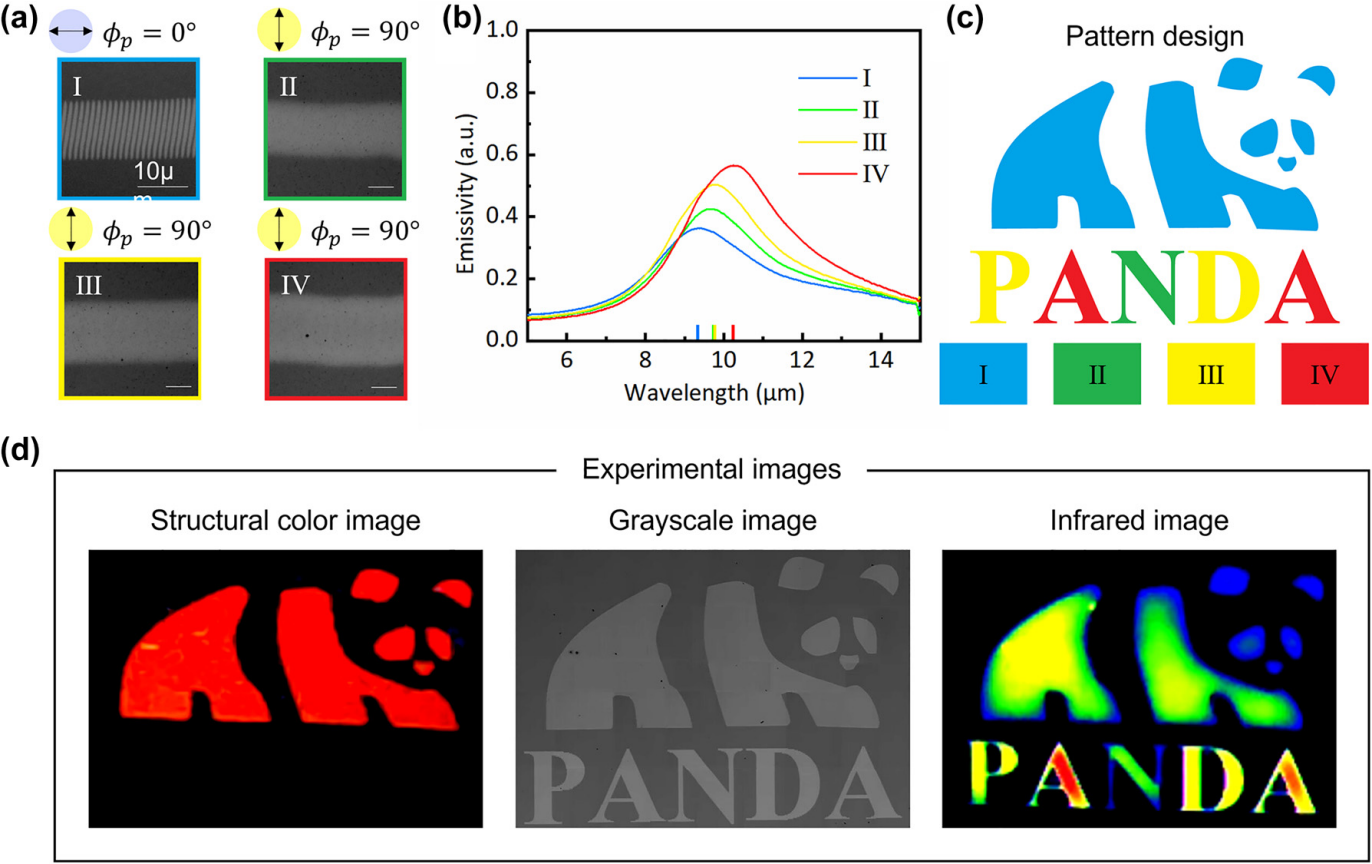
Experimental results of the visible-infrared-compatible anti-counterfeiting. (a) Experimental optical micrographs of GST surface with laser-scanned modified lines using four polarization-pulse energy combinations: I (0°, 49.2 nJ), II (90°, 71.3 nJ), III (90°, 128.5 nJ), and IV (90°, 175.1 nJ). (b) Emittance spectra of GST measured under the same four polarization-pulse energy combinations as in (a). (c) Diagram illustrating a steganographic panda pattern with different colors representing various fabrication parameters, denoted as I–IV. (d) Experimental images captured by the camera under strong illumination, optical microscope, and infrared camera, respectively.

We have used unpatterned GST thin films to directly optically write tunable gratings, achieving multilevel infrared camouflage. The laser-induced modified surface structures with structural gratings exhibit visible diffraction and IR emissivity properties. Subsequently, we conducted further studies for applications in visible-infrared-compatible image information storage and encryption. Figure 6a displays four types of laser-scanned modified lines on GST surfaces created under various polarization-pulse energy combinations: I corresponds to processing with a 0° polarization and 49.2 nJ energy, while II, III, and IV represent processing with 90° polarization and energy levels of 71.3 nJ, 128.5 nJ, and 175.1 nJ, respectively. Figure 6b illustrates the measured infrared emissivity spectra under the same polarization-pulse energy combinations as shown in Figure 6a. The region I of modified surface structures shown in Figure 6c of a panda pattern was written into a GST film by ps laser with polarization direction parallel to direct writing direction. And the region II–IV of fully crystallized structures shown in Figure 6c of the letters ‘P’, ‘A’, ‘N’, ‘D’, ‘A’ pattern was written by ps laser with polarization direction perpendicular to direct writing direction. The image, captured under strong illumination, is depicted in Figure 6d (left) and presents a red panda image with identifiable structural colors. Notably, the letters remained concealed due to the absence of geometric grating structures. Consequently, the panda image information was displayed while the letter information was encrypted. Additionally, the grating’s diffraction effect allowed us to achieve diverse colors such as green and blue by altering the incident and viewing angles. The image captured by the IR camera of the same sample is depicted in Figure 6d (right), showcasing four distinct IR levels with varying thermal emissivity for both the panda image and the letters. Three distinct crystallized structures, each with varying crystallinity induced by the ps laser as the pulse energy increased. In summary, we have successfully accomplished multilevel image data storage in the IR spectrum while simultaneously encrypting partial information in the visible spectrum.

## 3 Conclusions

In this work, we presented a one-step ultrafast laser writing method for dynamic and multilevel tuning of the infrared thermal emission on the GST film. In this approach, we used an ultrafast laser to directly write crystallization micro/nano resonant structures on amorphous GST films with specific properties. We achieved this by precisely controlling the laser’s polarization and pulse energy. Compared to traditional methods, this maskless approach enables the precise processing of arbitrary patterns. Meanwhile, this easy fabrication is vital for low-cost and mass applications. In addition, by direct induction of ultrafast laser to control the structures and properties of resonant units, the metasurface has visible-infrared compatible dual-band response. Moreover, the introduction of phase-change material GST enables dynamic control of both the visible structural color and the infrared thermal emission. We conducted experiments to demonstrate multilevel IR camouflage and the storage of visible-infrared compatible information using ultrafast laser-induced GST emitters. Additionally, we exhibited spatially selective dynamic control and multilevel continuous modulation of emissivity. These capabilities establish a stable and effective platform for security applications, including anti-counterfeiting and information security. Consequently, this work may open opportunities for further developments of multiband dynamic devices and novel applications such as IR data storage, encryption, camouflage, thermal management, and so on. Finally, we envision that a wider IR emissivity control range can be reached by combining different active materials, and the complete reversible phase-change process induced by ultrafast laser can be applied to GST IR emitters by designing a resonant cavity.

## 4 Experimental section

### 4.1 Sample preparation

Initially, amorphous GST thin films were deposited on Si (100) substrates using a radio-frequency magnetron sputtering system (MSP-300B, Beijing Chuangshi Weina Technology, China) with a high-purity stoichiometric target. The Si substrates were pre-cleaned with an ethanol solution and rinsed with deionized water under ultrasonic agitation to remove surface contaminants before sputtering. Sputtering was carried out at room temperature with an Ar gas flow, a pressure of 0.4 Pa, and a power of 30 W, maintaining a deposition rate of 40 nm/min. The film thickness was verified using a laser scanning confocal microscope (OLS5100, Olympus, Japan).

### 4.2 Ultrafast laser fabrication

To fabricate the nanogratings, we used a picosecond (ps) laser system (Huaray) that delivers Gaussian-shaped pulses with a pulse duration of 9.3 ps, a central wavelength of 1064 nm, and a repetition rate of 200 kHz. In this research, the laser pulse repetition rate was reduced to 1 kHz by using a pulse picker. The ps laser pulses were focused on the sample surface through an F-theta lens (*f* = 50 mm). Neutral-density filters were employed to enable variable adjustment of the laser pulse energy incident on the sample surface. A half-wave plate was used to change the polarization direction of the incident laser pulse. Samples were mounted one at a time on a computer-controlled three-axis moving stage (Aerotech A3200).

### 4.3 Sample characterization

The morphologies and microstructures of the fabricated samples were characterized using optical microscopy (BX51, Olympus, Japan), and atomic force microscopy (AFM; Dimension Edge PSS, Bruker, Germany). X-ray diffraction (XRD) patterns were obtained in the 10°–70° (2*θ*) angle range using an X-ray diffractometer (D/Max 2500 H, Rigaku, Japan). Infrared spectral data and spectral imaging were obtained using a Fourier transform infrared spectrometer (VERTEX 70v, Bruker, Germany). Infrared patterns were obtained in the 8–14 μm wavelength range using an on-line infrared thermograph (AT61P5DSHT, InfiRay, China).

### 4.4 Numerical simulations

Numerical simulations were performed using the commercial software Lumerical FDTD Solutions. Periodic boundary conditions were applied in the *x* and *y* directions for plane wave propagation, while perfectly matched layer boundaries were used in the *z*-direction.

## Supplementary Material

Supplementary Material Details

## References

[1] Xiao L. (2015). Fast adaptive thermal camouflage based on flexible VO_2_/graphene/CNT thin films. *Nano Lett.*.

[2] Salihoglu O. (2018). Graphene-based adaptive thermal camouflage. *Nano Lett.*.

[3] Guan Y. (2023). Mechanochromic photonic vitrimer thermal management device based on dynamic covalent bond. *Adv. Funct. Mater.*.

[4] Zhang Q. (2022). Bioinspired zero-energy thermal-management device based on visible and infrared thermochromism for all-season energy saving. *Proc. Natl. Acad. Sci. U. S. A.*.

[5] Lochbaum A. (2017). On-chip narrowband thermal emitter for mid-IR optical gas sensing. *ACS Photonics*.

[6] Shahsafi A. (2019). Temperature-independent thermal radiation. *Proc. Natl. Acad. Sci. U. S. A.*.

[7] Inoue T. (2014). Realization of dynamic thermal emission control. *Nat. Mater.*.

[8] Hu R. (2021). Thermal camouflaging metamaterials. *Mater. Today*.

[9] Zhu H. (2020). High-temperature infrared camouflage with efficient thermal management. *Light Sci. Appl.*.

[10] Qu Y. (2018). Thermal camouflage based on the phase-changing material GST. *Light Sci. Appl.*.

[11] Dan A. (2019). Temperature- and angle-dependent emissivity and thermal shock resistance of the W/WAIN/WAION/Al_2_O_3_-based spectrally selective absorber. *ACS Appl. Energy Mater.*.

[12] Xiao Y. (2019). Nanosecond mid-infrared pulse generation via modulated thermal emissivity. *Light Sci. Appl.*.

[13] Shao Z. (2022). All-solid-state proton-based tandem structures for fast-switching electrochromic devices. *Nat. Electron.*.

[14] Guo J. (2023). Fast-switching WO_3_-based electrochromic devices: design, fabrication, and applications. *Acc. Mater. Res.*.

[15] Tang K. (2021). Temperature-adaptive radiative coating for all-season household thermal regulation. *Science*.

[16] Tittl A. (2015). A switchable mid-infrared plasmonic perfect absorber with multispectral thermal imaging capability. *Adv. Mater.*.

[17] Ko J. H. (2023). Polarization-driven thermal emission regulator based on self-aligned GST nanocolumns. *iScience*.

[18] Zhao K. (2022). Ultrafast laser-induced integrated property-structure modulation of Ge_2_Sb_2_Te_5_ for multifunction and multilevel rewritable optical recording. *Nanophotonics*.

[19] Du K. (2018). Wavelength-tunable mid-infrared thermal emitters with a non-volatile phase changing material. *Nanoscale*.

[20] Hosseini P., Wright C. D., Bhaskaran H. (2014). An optoelectronic framework enabled by low-dimensional phase-change films. *Nature*.

[21] Han Z. H. (2020). Ultrafast temporal-spatial dynamics of amorphous-to-crystalline phase transition in Ge_2_Sb_2_Te_5_ thin film triggered by multiple femtosecond laser pulses irradiation. *Nanotechnology*.

[22] Zhang J. (2020). Near-infrared rewritable, non-volatile subwavelength absorber based on chalcogenide phase change materials. *Nanomaterials*.

[23] Kim D. H. (2023). Polarization-mediated multi-state infrared system for fine temperature regulation. *APL Photonics*.

[24] Ko J. H. (2022). A review of tunable photonics: optically active materials and applications from visible to terahertz. *iScience*.

[25] Karvounis A. (2016). All-dielectric phase-change reconfigurable metasurface. *Appl. Phys. Lett.*.

[26] Kim Y., Kim C., Lee M. (2022). Parallel laser printing of a thermal emission pattern in a phase-change thin film cavity for infrared camouflage and security. *Laser Photon. Rev.*.

[27] Raeis-Hosseini N., Rho J. (2017). Metasurfaces based on phase-change material as a reconfigurable platform for multifunctional devices. *Materials*.

[28] Wang J., Wang L., Liu J. (2020). Overview of phase-change materials based photonic devices. *IEEE Access*.

[29] Kolobov A. V. (2004). Understanding the phase-change mechanism of rewritable optical media. *Nat. Mater.*.

[30] Njoroge W. K., Wöltgens H. W., Wuttig M. (2002). Density changes upon crystallization of Ge_2_Sb_2.04_Te_4.74_films. *J. Vac. Sci. Technol. A*.

[31] Li P. (2016). Reversible optical switching of highly confined phonon-polaritons with an ultrathin phase-change material. *Nat. Mater.*.

[32] Michel A.-K. U. (2019). Advanced optical programming of individual meta-atoms beyond the effective medium approach. *Adv. Mater.*.

[33] Lian Y. (2023). Ultrafast quasi-three-dimensional imaging. *Int. J. Extrem. Manuf.*.

[34] Wang Q. (2017). Reconfigurable phase-change photomask for grayscale photolithography. *Appl. Phys. Lett.*.

[35] Guosheng Z., Fauchet P. M., Siegman A. E. (1982). Growth of spontaneous periodic surface structures on solids during laser illumination. *Phys. Rev. B*.

[36] Xu Z. (2018). Optical constants acquisition and phase change properties of Ge_2_Sb_2_Te_5_ thin films based on spectroscopy. *RSC Adv.*.

