# Supplementary material for: Metasurface with all-optical tunability for spatially-resolved and multilevel thermal radiation
Source: Nanophotonics. 2024 Mar 13;13(9):1645–55. doi: 10.1515/nanoph-2024-0005 (PMC11636409; doi:10.1515/nanoph-2024-0005)
Supplement: Supplementary file 1 — Supplementary Material Details [file j_nanoph-2024-0005_suppl_001.docx]

**Supporting Information**

Shuhui Jiao, Kang Zhao, Jianhui Jiang, Kailin Zhao, Qin Guo, Jingbo Wang, Yansong Zhang, Gang Chen, Qian Cheng, Pei Zuo, and Weina Han*

Metasurface with all-optical tunability for spatially-resolved and multilevel thermal radiation


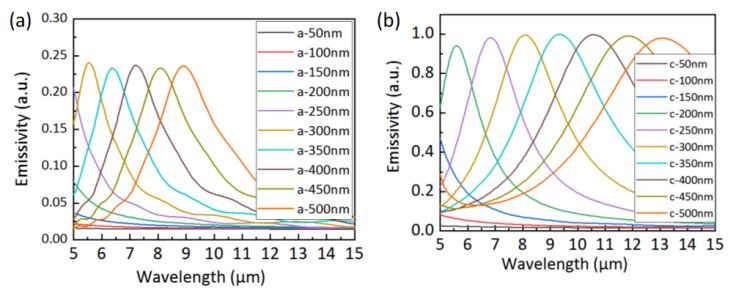


**Figure S1:** (a) Emissivity spectra of multilayer film structures based on a-GST layers of different film thicknesses. (b) Emissivity spectra of multilayer film structures based on c-GST layers of different film thicknesses.

Figure S1 shows the influence of GST layer on spectral modulation. With the increase of film thickness, the peak absorption rate of GST-based multilayer film structure increases, and the strength remains unchanged. This is due to the fact that the top layer of phase change material forms a Fabry-Parro resonator with the middle layer of metal material, and the position of its formant will be redshifted with the increase of film thickness. At the same time, because a-GST is almost transparent in the mid-infrared band, most of the light passing through the a-GST layer is not absorbed by it. Therefore, the peak absorption intensity of a-GST is significantly lower than that of c-GST when the film thickness is the same. From the above analysis, it can be seen that when the thickness of GST increases, the peak position of its absorption rate will also increase. Therefore, wavelength optional spectral regulation can be performed according to the film thickness, which also provides a strong basis for the preparation of samples used in the later experiments of this study.


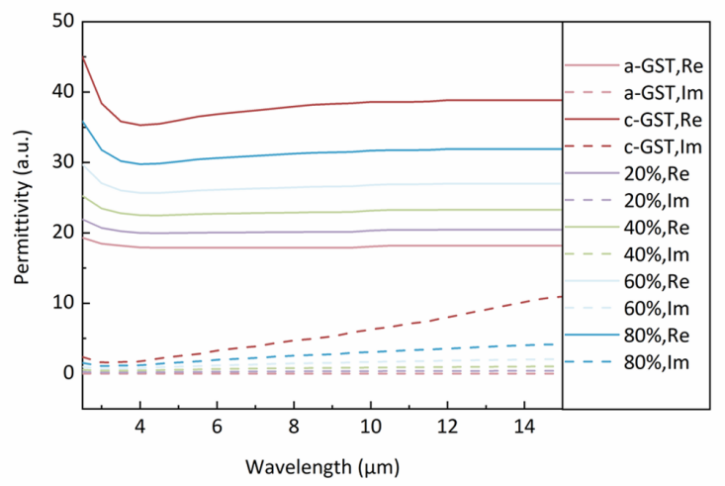


**Figure S2:** The function curve of permittivity of GST with different crystallinity varies with wavelength.

Figure S2 shows the dielectric constant of GST with different crystallinity was calculated by Lorentz-Lorenz formula. The crystallinity of aGST is 0, and the crystallinity of cGST is 1. The solid line represents the size of the real part of the permittivity, the dashed line represents the size of the imaginary part of the permittivity, and the crystallinity ranges from 0 to 100% in steps of 20%. It can be seen from the figure that with the change of crystallinity, both the real and imaginary parts of GST permittivity change greatly. Through the above measurement, extraction and calculation of the refractive index or dielectric constant of GST materials, the information of GST materials in different bands and different crystallinity can be used for FDTD Solutions simulation, which provides data support for the subsequent simulation of material property changes for metasurface spectral regulation and electromagnetic field regulation.


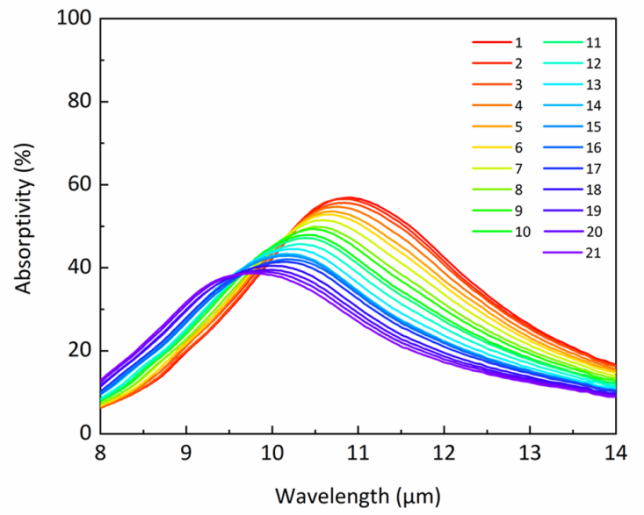


**Figure S3:** Continuous infrared emissivity spectra.

Figure S3 shows the 21 levels of emissivity spectrum used to write the pattern in Figure 4c. Each column of the QR code pattern corresponds to these 21 levels. After calculation, the difference between adjacent average emissivity of these 21 orders in the infrared atmospheric window of 8-14 microns is 0.0014, 0.0076, 0.0037, 0.0053, 0.0065, 0.0067, 0.0108, 0.0025, 0.0082, 0.0046, 0.0082, 0.0063, 0.0058, 0.0110, 0.0045, 0.0023, 0.0054, 0.0058, 0.0021, 0.0036, respectively. Among them, the minimum adjustment accuracy is 0.0014. In this way, almost continuous arbitrary emissivity regulation can be obtained.


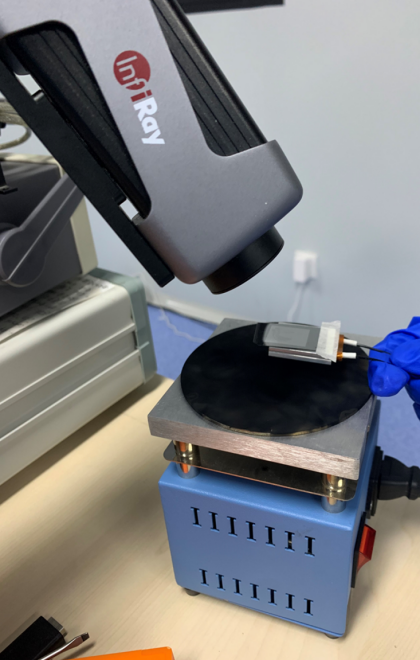


**Figure S4:** Simulation of thermal camouflage device diagram.

The device comprises five components (Figure S4): an infrared thermal imager, the test sample, a fixed-temperature micro heater, a self-made black body, and a numerically controlled variable-temperature heating stage. Among them, the constant temperature heating table is composed of a heating resistance sheet and a power supply. When powered, the heating resistance sheet maintains a temperature of 80 °C. In this experiment, a stainless steel disc with a layer of black soot on its surface was employed to simulate the environmental background. The soot was evenly distributed on the stainless steel sample's surface by briefly exposing it to a candle's flame for 30 seconds. Black soot is frequently used as a reference for approximating a blackbody due to its high emissivity, which is independent of wavelength (approximately 0.97). The self-made black body was positioned on the numerically controlled variable-temperature heating stage, allowing us to adjust the background temperature in the range of 25 °C to 60 °C. The sample is affixed to the micro heater's surface, maintaining the sample's actual temperature at 80 °C. The uppermost infrared thermal imager records the sample's multilevel camouflage response to temperature variations.


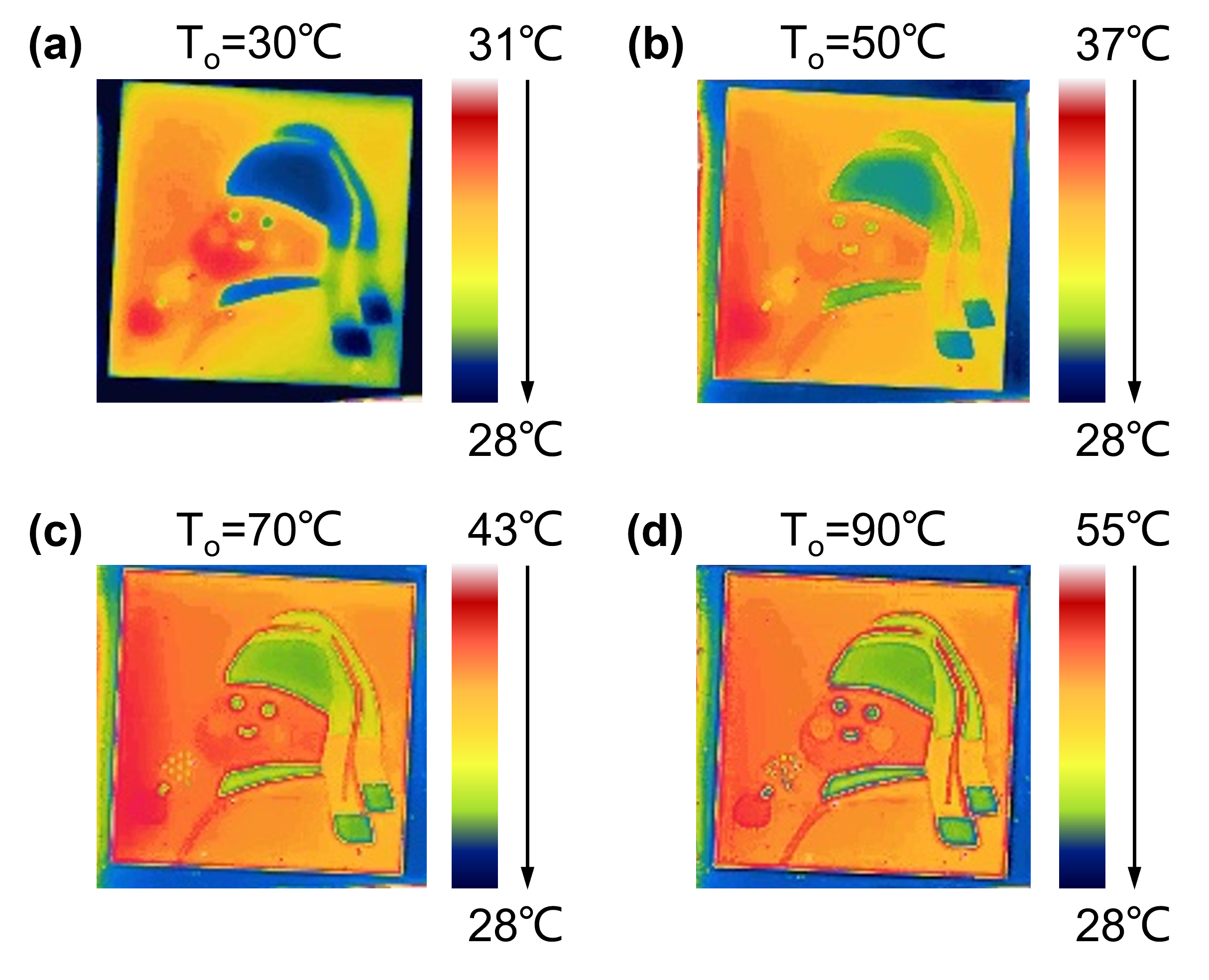


**Figure S5:** Thermal images of multilevel thermal device with actual temperature of (a) 30 °C, (b) 50 °C, (c) 70 °C, and (d) 90 °C, respectively.

In this experiment, we fixed the sample on a heating stage to change its actual temperature from 30 °C to 90 °C. It is evident that the device can work with high-contrast spatial emissivity when the temperature of itself remains a relatively low temperature 30 °C or a relatively high temperature 90 °C. So we can say that the temperature range of the device to work can be from room temperature to 168 °C (phase change temperature of GST). However, the radiation temperature range of different levels of the device is different when the actual temperature of the object To is different. And this radiation temperature range (28 °C to 31 °C) is narrow when To is relatively low. And the radiation temperature range (28 °C to 55 °C) is wide when To is relatively high.


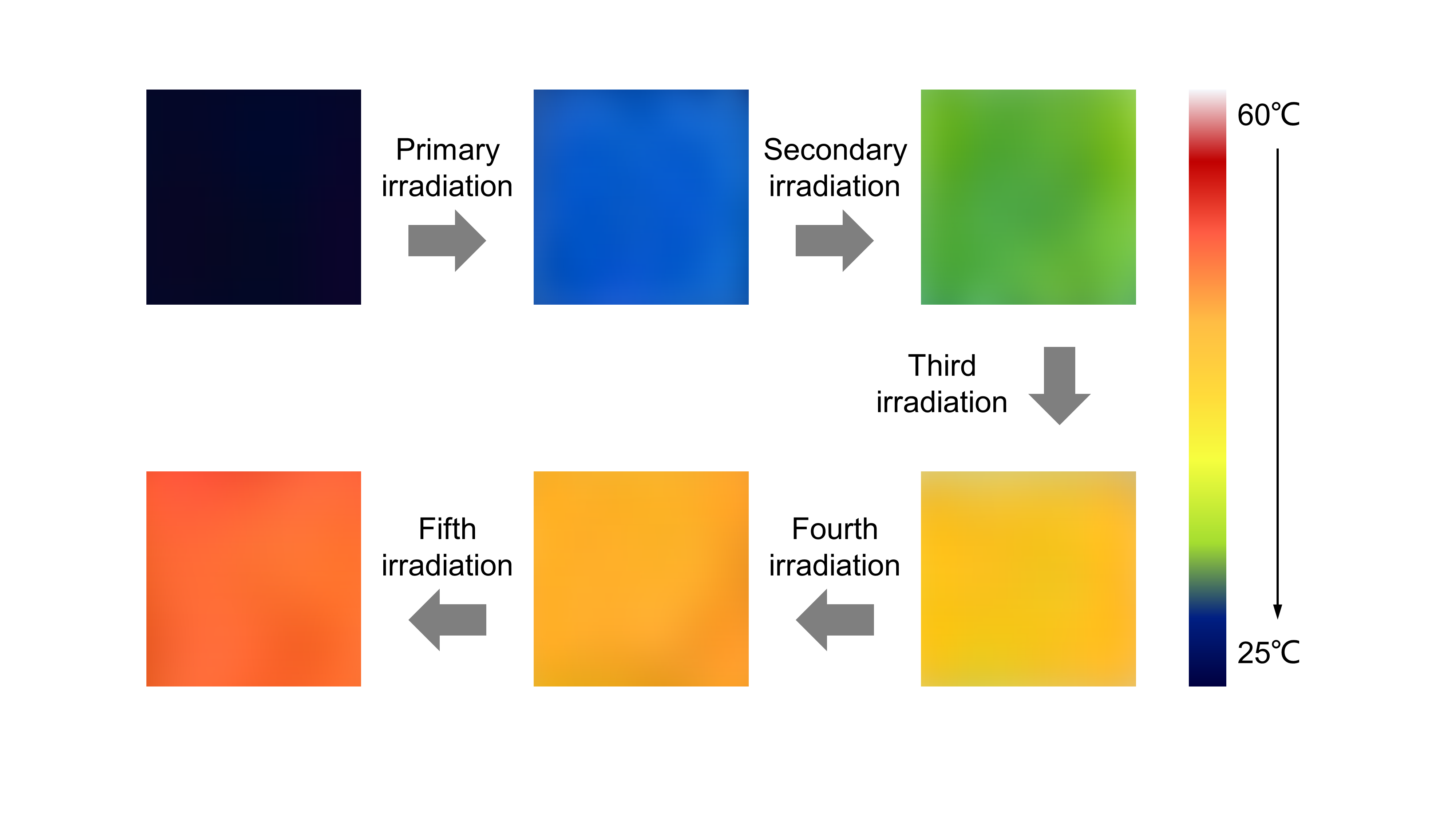


**Figure S6:** Thermal images of the GST emitter under different laser irradiation times.

We can realize dynamic switching between different levels of the device by multiple irradiation of ultrafast laser. This also proves that we can achieve the precise control of multi-level thermal radiation as we thought. The precision of the modulation is not as continuous as that of fabrication, because modulation is carried out on the existing structure. But we can achieve a steady and precise modulation of thermal emission.


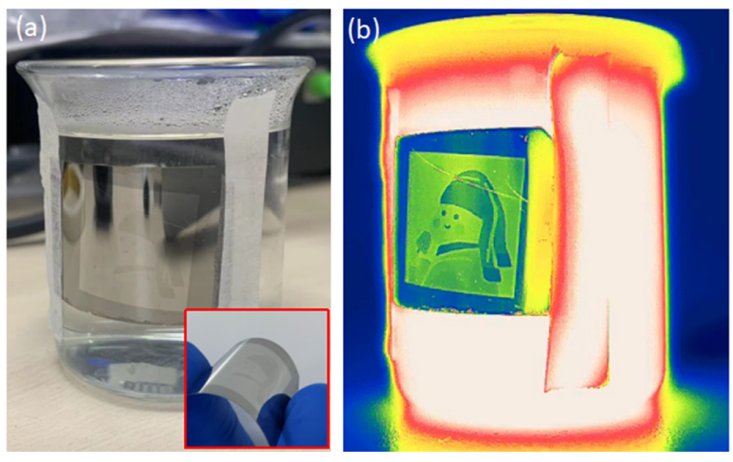


**Figure S7:** Device flexible effect display.

In this investigation, we fabricated multilayer film structures, consisting of a 100nm-thick metallic aluminum layer and a 350nm-thick amorphous GST film, on a flexible substrate (polyester film, PET) using a magnetron sputtering instrument. Furthermore, we created multi-stage cartoon patterns on the sample surface by employing an ultrafast laser, as depicted in Figure S5. The flexible device is affixed to the surface of a cup containing hot water (Figure S5a), and its imaging is presented in Figure S5b through an infrared thermal imager. Evidently, the sample conceals the actual temperature of the water cup, thereby altering the radiative temperature of the object's surface. Additionally, multiple patterns are observable on the sample's surface due to varying average emissivity. Simultaneously, the metasurface device is capable of adjusting radiation temperature to match the background temperatures, making it useful for infrared information concealment.

**Movie S1.** Multilevel IR camouflage at varying background temperatures.

**Movie S2.** IR flexible device on a cup containing gradually cooling hot water.
